# IgG Responses to Porins and Lipopolysaccharide within an Outer Membrane-Based Vaccine against Nontyphoidal *Salmonella* Develop at Discordant Rates

**DOI:** 10.1128/mBio.02379-17

**Published:** 2018-03-06

**Authors:** Anna E. Schager, C. Coral Dominguez-Medina, Francesca Necchi, Francesca Micoli, Yun Shan Goh, Margaret Goodall, Adriana Flores-Langarica, Saeeda Bobat, Charlotte N. L. Cook, Melissa Arcuri, Arianna Marini, Lloyd D. W. King, Faye C. Morris, Graham Anderson, Kai-Michael Toellner, Ian R. Henderson, Constantino López-Macías, Calman A. MacLennan, Adam F. Cunningham

**Affiliations:** a Institute for Microbiology and Infection, School of Immunology and Immunotherapy, Institute for Biomedical Research, University of Birmingham, Birmingham, United Kingdom; b GSK Vaccines Institute for Global Health, Siena, Italy; c Medical Research Unit on Immunochemistry, National Medical Center “SigloXXI,” Specialties Hospital, Mexican Institute for Social Security (IMSS), Mexico City, Mexico; Jenner Institute; Max Planck Institute for Infection Biology

**Keywords:** antibodies, B-cell responses, infection, outer membrane vesicles, *Salmonella*, vaccines

## Abstract

Antibodies acquired after vaccination or natural infection with Gram-negative bacteria, such as invasive *Salmonella enterica* serovar Typhimurium, can protect against disease. Immunization with naturally shed outer membrane vesicles from Gram-negative bacteria is being studied for its potential to protect against many infections, since antigens within vesicles maintain their natural conformation and orientation. Shedding can be enhanced through genetic modification, and the resulting particles, generalized modules for membrane antigens (GMMA), not only offer potential as vaccines but also can facilitate the study of B-cell responses to bacterial antigens. Here we show that the response to immunization with GMMA from *S*. Typhimurium (STmGMMA) provides B-cell-dependent protection and induces antibodies to two immunodominant antigens, lipopolysaccharide (LPS) and porins. Antibodies to LPS O antigen (O-Ag) markedly enhance protection in the spleen, but this effect is less marked in the liver. Strikingly, IgG responses to LPS and porins develop with distinct kinetics. In the first week after immunization, there is a dramatic T-cell-independent B1b-cell-associated induction of all IgG isotypes, except IgG1, to porins but not to LPS. In contrast, production of IgG1 to either antigen was delayed and T cell dependent. Nevertheless, after 1 month, cells in the bone marrow secreting IgG against porins or LPS were present at a similar frequency. Unexpectedly, immunization with O-Ag-deficient STmGMMA did not substantially enhance the anti-porin response. Therefore, IgG switching to all antigens does not develop synchronously within the same complex and so the rate of IgG switching to a single component does not necessarily reflect its frequency within the antigenic complex.

## IMPORTANCE

Vaccines save millions of lives, yet for some infections there are none. This includes some types of *Salmonella* infections, killing hundreds of thousands of people annually. We show how a new type of vaccine, called GMMA, that is made from blebs shed from the *Salmonella* cell wall, works to protect against infection in mice by inducing host proteins (antibodies) specifically recognizing bacterial components (antigens). The rate of development of IgG antibody to antigens within GMMA occurred with different kinetics. However, the antibody response to GMMA persists and is likely to provide prolonged protection for those who need it. These results help show how antibody responses to bacterial antigens develop and how vaccines like GMMA can work and help prevent infection.

## INTRODUCTION

Bacterial infections remain a serious threat to human and veterinary health. Novel, cost-effective vaccination strategies are needed for use in resource-limited regions of the world, such as sub-Saharan Africa. A promising approach to generate vaccines against Gram-negative bacteria is to use native outer membrane vesicles (NOMVs), which are blebs of outer membrane naturally shed by bacteria. Key advantages of NOMVs include their potential safety for use in humans (1), enrichment for surface antigens that are often recognized by B cells, and maintenance of these antigens in their natural conformation and orientation (2). Furthermore, these nano-sized, nonviable antigens can be used in immunocompromised individuals and overcome the potential risks of infection associated with the use of live, attenuated vaccines in such populations.

To enhance the production of OMVs from Gram-negative bacteria without using detergents, mutations can be introduced that result in hyperblebbing. This avoids the potential for detergents to alter the conformation of some antigens within the particles and to extract some lipoproteins from them. Thus, high yields can be obtained from bacteria in which the Tol-Pal pathway is disrupted by deletion of *tolR* (3). The resulting particles are similar to NOMVs and known as generalized modules of membrane antigens (GMMA). GMMA and NOMVs have been assessed as vaccine platforms to induce protective immunity against several Gram-negative bacterial pathogens such as *Escherichia coli*, *Shigella*, *Salmonella*, and *Neisseria* (1, 3–19). Invasive nontyphoidal *Salmonella* (iNTS) infections are a serious health concern and are estimated to kill over 650,000 people annually worldwide (20). Two *Salmonella enterica* serovars, Typhimurium and Enteritidis, are predominantly associated with iNTS disease in children under 5 years old in sub-Saharan Africa, and iNTS infections are a serious problem in individuals of any age with HIV infection. Despite this, there is no vaccine against iNTS infections that is licensed for use in humans (21–23). NOMVs have previously been shown to have potential against experimental iNTS infections (8), and one likely mechanism for their mode of action is the induction of a protective antibody (Ab) that can play a role in the control of such infections (24). Antibodies to several *S*. Typhimurium antigens have been shown to be protective, including the O antigen (O-Ag) of the lipopolysaccharide (LPS) molecule (25–30) and porins (31–35), including OmpD (33). While antibodies to LPS and porins are detectable after natural infection, little is known about the relative kinetics of their induction, particularly in the context of OMVs and GMMA. Furthermore, the relative persistence of responses to these antigens has received limited attention. This is important to know, as both the induction and the maintenance of responses to antigen are key requisites of any vaccine that provides long-lasting immunity.

In this study, mice were immunized with GMMA from *S*. Typhimurium (STmGMMA) and the relative kinetics of the induction of antibodies of different IgG isotypes to porins and LPS were addressed. The results show that IgG responses to LPS develop more slowly than IgG responses to porins, with a paucity of IgG1 to both antigens detected, compared to other IgG isotypes, until the second week after immunization. Unexpectedly, the induction of IgG of all isotypes except IgG1 was independent of T cells, possibly reflecting the B1b-cell response to STmGMMA observed. Collectively, these data show that B-cell responses to all antigens do not develop in parallel within the same GMMA antigenic complex and that the extent of the early IgG response does not necessarily reflect the relative abundance of an antigen within a complex.

## RESULTS

### Immunization with STmGMMA induces protection that is dependent upon B cells

**FIG 1:**
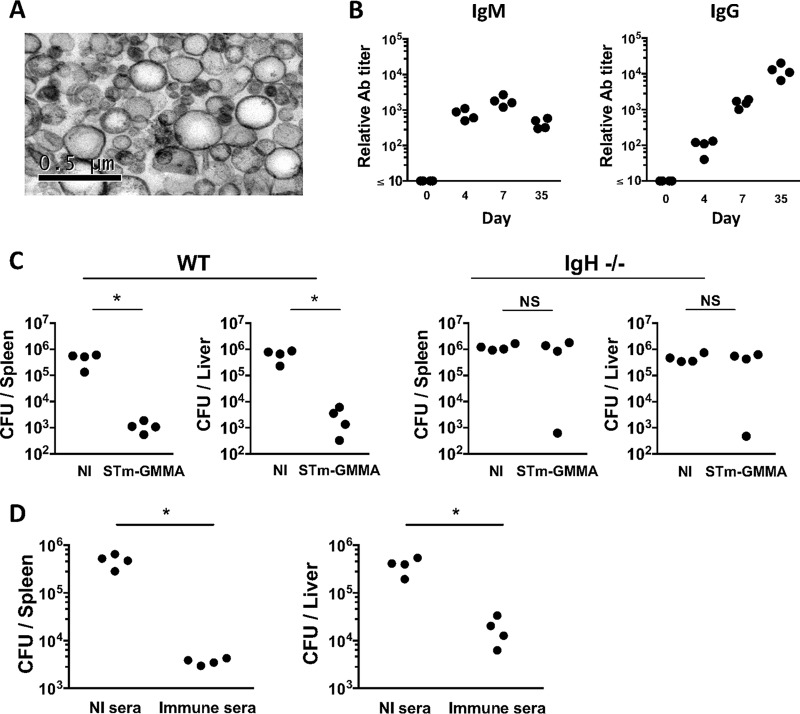
STmGMMA induce antibodies that can protect against infection. (A) Electron microscopy of STmGMMA. STmGMMA were purified from *S*. Typhimurium Δ*tolR* culture supernatant, prepared for staining, and visualized by TEM. (B) Mice were immunized i.p. with 1 μg of STmGMMA for the times indicated, and serum samples were subjected to ELISA for anti-STmGMMA IgM (left) and IgG (right). Each dot represents one serum sample. (C) WT (left) or B-cell-deficient (IgH^−/−^) (right) mice immunized once i.p. with 1 μg of STmGMMA for 14 days were infected i.p. with 5 × 10^5^ CFU of *S*. Typhimurium SL3261, and bacteria in the spleen (left) and liver (right) were enumerated 5 days postinfection. Each dot represents one mouse, and graphs are representative of two independent experiments. (D) Naive WT mice were infected i.p. with 5 × 10^5^ CFU of *S*. Typhimurium SL3261 that were preopsonized with serum from nonimmunized mice or serum from mice immunized with STmGMMA for 35 days. Bacteria in the spleen (left) and liver (right) were enumerated at 24 h postinfection. Each dot represents one mouse, and results representative of two independent experiments are shown. The Mann-Whitney U test was used to determine significant differences between groups linked by bars. NI, nonimmunized; NS, nonsignificant;*, *P* ≤ 0.05.

STmGMMA were purified from the supernatants of cultures of *S*. Typhimurium SL1418 Δ*tolR*. Transmission electron microscopy (TEM) showed that STmGMMA are spherical particles of various sizes, with diameters in the range of approximately 20 to 250 nm, similar to previous reports describing GMMA (3, 5, 6) and OMVs (2, 8) (Fig. 1A). After immunization of C57BL/6 mice intraperitoneally (i.p.) with 1 μg of STmGMMA, there was a rapid induction of IgM and IgG, with both detectable by day 4, and while IgM titers remained relatively stable, IgG titers continued rising over the following weeks (Fig. 1B). Mice immunized with STmGMMA for 14 days showed a >100-fold reduction in bacterial burdens in the spleen and liver when challenged i.p. with attenuated *S*. Typhimurium SL3261. In contrast, B-cell-deficient mice immunized in the same way did not have any significant bacterial burden reduction at these sites (Fig. 1C). Experiments where nonimmunized mice were infected for 24 h with bacteria that had been opsonized with serum from nonimmunized or STmGMMA-immunized mice showed that the presence of anti-STmGMMA Ab was sufficient to reduce bacterial colonization of the spleen and liver (Fig. 1D). Therefore, immunization with STmGMMA induces rapid IgM and IgG responses and confers protection that is dependent upon B cells.

### IgG switching and GC are induced rapidly after immunization with STmGMMA.

**FIG 2:**
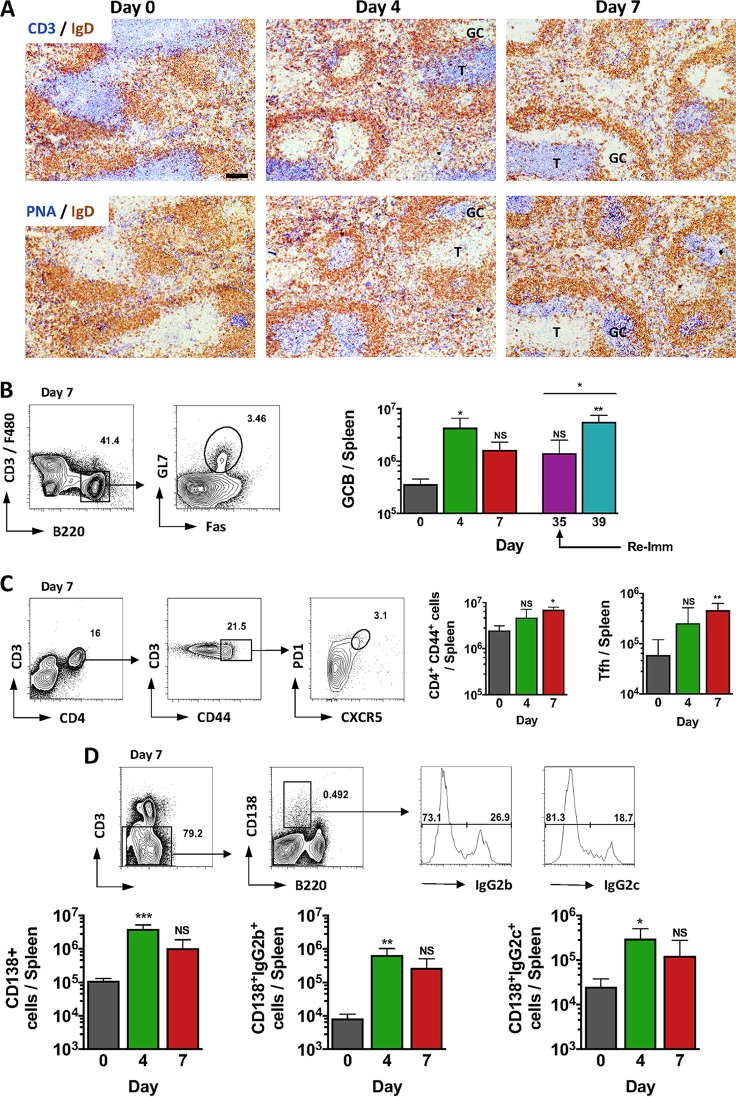
STmGMMA induce rapid and extensive B- and T-cell responses after immunization. Mice were immunized i.p. with 1 μg of STmGMMA for the times indicated in days following primary immunization. (A) Representative photomicrographs of spleen sections from nonimmunized mice and mice immunized with STmGMMA stained for IgD (brown) and CD3 (blue; top row) or PNA (blue; bottom row) for nonimmunized WT mice (left) and mice immunized for 4 (middle) or 7 (right) days. Scale bar, 100 μm. (B to D) Representative FACS plots demonstrating gating and enumeration of cells (all from mice immunized for 7 days) from spleens stained for GC B (GCB) cells (CD3^−^ F4/80-B220^+^ is Fas and GL7 positive) (day 39 indicates mice boosted at day 35) (B), splenic activated T cells (CD3^+^ CD44^+^ CD4^+^) (left graph) and Tfh cells (CD3^+^ CD44^+^ CD4^+^ is CXCR5^+^ PD1^+^) (right graph) from a mouse immunized for 7 days (C), and plasma cells (CD3^−^ B220^lo^ CD138^+^) (left graph), including plasma cells stained positive for intracellular IgG2b (middle graph) or IgG2c (right graph) (D). Graphs show the mean value and standard deviation of four mice per group, and the results are representative of two independent experiments. T, T zone; Re-imm, reimmunization. Statistical analysis was performed with the Kruskal-Wallis test and Dunn’s multiple-comparison test, with time zero as the reference time point. The Mann-Whitney U test was used to compare the day 35 and 39 time points in panel B. NS, nonsignificant; *, *P* ≤ 0.05; **, *P* ≤ 0.01; ***, *P* ≤ 0.001.

Since B cells were important for the protection afforded by STmGMMA immunization, the nature of the B-cell response was examined in more depth. Mice immunized i.p. with 1 μg of STmGMMA showed a clear induction of splenic germinal centers (GC) and GC B cells at 4 days after immunization (Fig. 2A and B). Numbers of GC B cells increased further after a second immunization with 1 μg of STmGMMA (Fig. 2B). Indeed, immunization induced a rapid increase in activated CD4^+^ T cells in the spleen by day 7, including a 3-fold increase in T follicular helper-like (Tfh) cells (Fig. 2C). Accompanying these features was an increase in the numbers of plasma cells, including cells that had switched to IgG2b and IgG2c (Fig. 2D). Taken together, these results show that STmGMMA induce a rapid and extensive T- and B-cell response.

### Ab responses to LPS and porins within STmGMMA develop with distinct kinetics.

**FIG 3:**
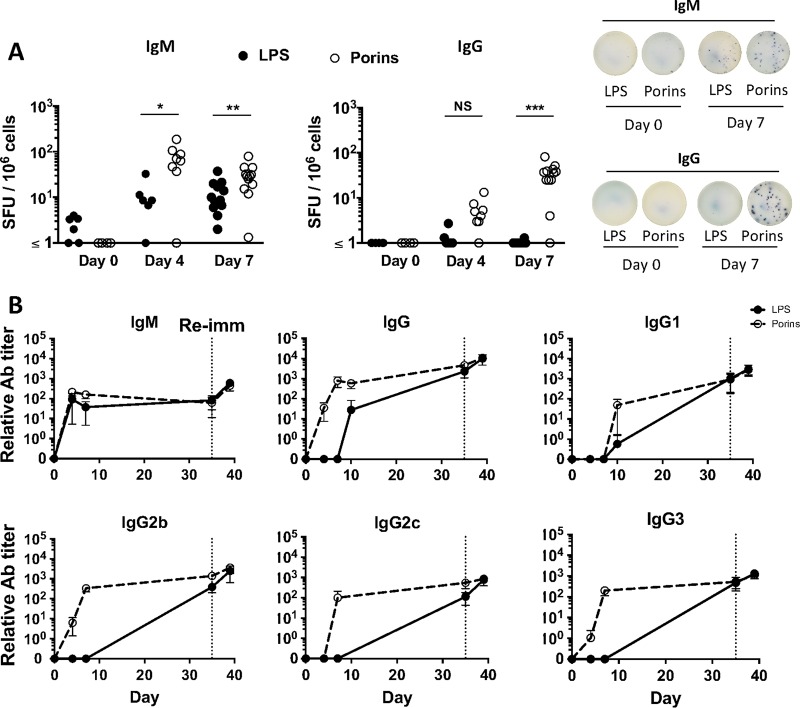
Nonparallel Ab responses to LPS and porins develop after immunization with STmGMMA. WT mice were immunized i.p. with 1 μg of GMMA for the times indicated. (A) B-cell ELISPOT assay used to assess IgM^+^ (left) and IgG^+^ (right) ASC against LPS (closed circles) and porins (open circles) in spleens 4 and 7 days after immunization. Numbers of spot-forming units (SFU) per million splenocytes are depicted in the graphs alongside representative pictures of wells after development. Each circle represents one mouse, and pooled results of three experiments are shown, except for the day 4 results, which are from two experiments with at least three mice per group. (B) Relative Ab titers, as determined by ELISA, in serum samples against LPS (closed circles, solid line) or porins (open circles, dashed line) from mice immunized with STmGMMA for the times indicated. Each circle represents the mean value of four mice ± the standard deviation. The dotted vertical line represents the time point of reimmunization (Re-imm). The Mann-Whitney U test with Bonferroni correction for multiple comparisons was used to determine significant differences between groups linked by bars. NS, nonsignificant; *, *P* ≤ 0.05; **, *P* ≤ 0.01; ***, *P* ≤ 0.001.

The rapid IgG response to STmGMMA observed led us to question whether this was induced similarly to multiple antigens within the particles or if responses within the same antigenic complex developed with distinct kinetics. To evaluate this, the responses to two immunodominant antigens, LPS and porins, were examined. Assessment of the B-cell response by enzyme-linked immunosorbent spot (ELISPOT) assay was used to assess antigen-specific IgM- and IgG-producing cells in the spleen at different time points following immunization with 1 μg of STmGMMA. IgM^+^ Ab-secreting cells (ASC) secreting Abs specific to both antigens were detected in the spleen at days 4 and 7 after immunization, albeit at higher frequencies for porins than for LPS. In contrast, IgG^+^ ASC specific to LPS were largely absent at both days 4 and 7 postimmunization, whereas IgG^+^ ASC specific to porins were readily detectable, and at an increasing frequency, at these two times (Fig. 3A). This nonparallel response was also reflected in the titers of Abs to both antigens in serum (Fig. 3B). IgG binding to LPS was not inhibited because of the presence of anti-LPS IgM antibodies, since depletion of IgM from serum samples with a specific monoclonal Ab (MAb) did not result in the detection of higher anti-LPS IgG titers (see Fig. S1 in the supplemental material). Enzyme-linked immunosorbent assays (ELISAs) were performed with serum samples from mice immunized with STmGMMA for 7 days or boosted for 4 days and digested with pepsin to generate F(ab′)_2_, Fab, and Fv fragments of IgM. These experiments showed that these fragments could still bind to LPS and porins, but the signal detected was weaker than that detected for IgM in undigested serum samples (Fig. S2). Thus, serum IgG to porins is detectable for days before IgG against LPS is detected. Analysis showed that at day 7, all subtypes except IgG1 against porins are present. Despite this early variability, by day 35, Abs of all subtypes to both LPS and porins were readily found (Fig. 3B). Therefore, although the host can clearly respond to multiple component antigens within a single antigenic complex, responses to the individual antigens present within GMMA can develop with distinct kinetics.

10.1128/mBio.02379-17.1 FIG S1  Depletion of IgM from STmGMMA-specific serum samples does not enhance anti-LPS IgG binding. Serum samples from individual mice immunized with STmGMMA for 7 or 35 days were split into two aliquots, one of which was depleted of IgM by mixing with anti-mouse IgM Ab coupled to Sepharose beads. These serum samples were then examined by ELISA for relative levels of total IgM (left) or IgG (right) antibodies (A), relative levels of anti-LPS and anti-porin IgM antibodies (B and C, respectively), and relative levels of anti-LPS and anti-porin IgG antibodies (D and E, respectively). Plots show the average OD at each dilution of serum from each group ± the standard deviation (*n* = 4 for day 7; *n* = 3 for day 35). Download FIG S1, TIF file, 0.1 MB. Copyright © 2018 Schager et al. 2018 Schager et al. This content is distributed under the terms of the Creative Commons Attribution 4.0 International license.

10.1128/mBio.02379-17.2 FIG S2  Binding of IgM and F(ab′)_2_, Fab, and Fv fragments to LPS and porins. Serum samples from individual mice immunized with STmGMMA for 7 days or boosted for 4 days after priming 35 days earlier were split into two aliquots, one of which was digested with pepsin for 24 h. Anti-LPS and anti-porin IgM antibody binding by digested (red lines) and undigested (blue lines) serum samples was assessed by ELISA. Graphs show the average OD_405_ ± the standard deviation at each dilution of serum from groups of three or four mice. Download FIG S2, TIF file, 0.3 MB. Copyright © 2018 Schager et al. 2018 Schager et al. This content is distributed under the terms of the Creative Commons Attribution 4.0 International license.

### STmGMMA induce T-cell-independent switching to all IgG isotypes except IgG1.

**FIG 4:**
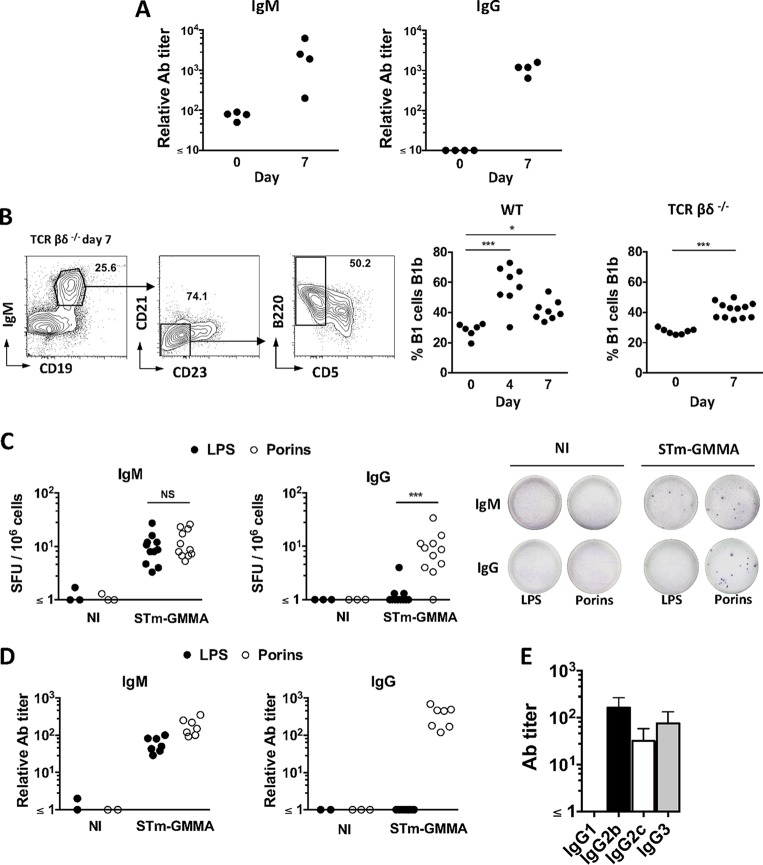
STmGMMA induce an early T-cell-independent anti-porin IgG response that is associated with B1b cells. (A) T-cell-deficient (TCRβδ^−/−^) mice were immunized i.p. with 1 μg of STmGMMA for 7 days, and serum samples were analyzed by ELISA for anti-STmGMMA IgM (left) and IgG (right). Each dot represents one serum sample. (B) WT or TCRβδ^−/−^ mice were immunized with 1 μg of STmGMMA for the times stated, and representative FACS gating for B1b cells from peritoneal cavity flushes is shown. B1 cells were identified as IgM^+^ CD19^+^ CD21^lo^ CD23^lo^, and B1b cells were identified as B1 cells that were CD5^−^ B220^+^ (representative FACS plots to demonstrate gating are from TCRβδ^−/−^ mice immunized for 7 days). Frequencies of B1b cells in B1 cells of WT (left) and TCRβδ^−/−^ (right) mice at the postimmunization time points indicated are shown in the graphs. Each dot represents one mouse, and pooled results of two experiments are shown. (C) IgM^+^ (left) and IgG^+^ (right) ASC against LPS (closed circles) or porins (open circles) in the spleens of TCRβδ^−/−^ mice immunized with STmGMMA for 7 days identified by B-cell ELISPOT assay. Numbers of spot-forming units (SFU) per million splenocytes are depicted in the graphs alongside representative pictures of wells after development. Each dot represents one mouse, and pooled results of multiple experiments are shown. (D) TCRβδ^−/−^ mice were immunized with STmGMMA for 7 days, and titers of IgM (left) and IgG (right) against LPS (closed circles) or porins (open circles) in serum samples were analyzed by ELISA. Each dot represents one serum sample, and the results are representative of two independent experiments. (E) TCRβδ^−/−^ mice were immunized with STmGMMA for 7 days, and IgG isotypes against porins in serum samples were analyzed by ELISA. Graphs show the mean values and standard deviations of groups of four mice. NI, nonimmunized. Statistical analysis was performed with the Kruskal-Wallis test and Dunn’s multiple-comparison test, with time zero as the reference time point for the left graph in panel B, and the Mann-Whitney U test was used for the right graph in panel B and for panel C. NS, nonsignificant; **, *P* ≤ 0.01; ***, *P* ≤ 0.001.

Although a humoral response associated with T-cell activation is seen after STmGMMA immunization, the surprisingly rapid and complex Ab response suggested that the response may include a T-cell-independent component. We therefore immunized T-cell-deficient mice with STmGMMA and determined the titers of IgM and IgG to STmGMMA in serum 7 days later. Both IgM and IgG were induced by 7 days after immunization in the absence of T cells (Fig. 4A). The T-cell-independent response to purified *S*. Typhimurium porins involves B1b cells (33). To establish if immunization with STmGMMA induced a B1b cell response, wild-type (WT) and T-cell-deficient mice were immunized with STmGMMA and the proportion of peritoneal B1 cells that are B1b cells was determined at 4 and 7 days after immunization. The proportion of B1b cells increased after STmGMMA immunization in both WT and T-cell-deficient mice, indicating that STmGMMA induce B1b responses (Fig. 4B). Assessment of responses by ELISPOT assay and ELISA 7 days after immunization showed that IgM to LPS and porins was induced in T-cell-deficient mice (Fig. 4C and D). Surprisingly, assessment of the specific responses to LPS and porins by ELISPOT assay and serology showed that in T-cell-deficient mice, there was a selective induction of antigen-specific IgG to porins, but not LPS, and that all IgG isotypes were induced at days 7 and 14, with the exception of IgG1 (representative results for day 7 are shown in Fig. 4C to E). Similar results were also observed at day 14, including a failure to detect antigen-specific IgG1. Therefore, immunization of mice lacking T cells shows that STmGMMA induce a strong T-cell-independent response and reveals a key role for T cells in the promotion of switching to IgG1.

### STmGMMA induce long-term responses to both LPS and porins.

**FIG 5:**
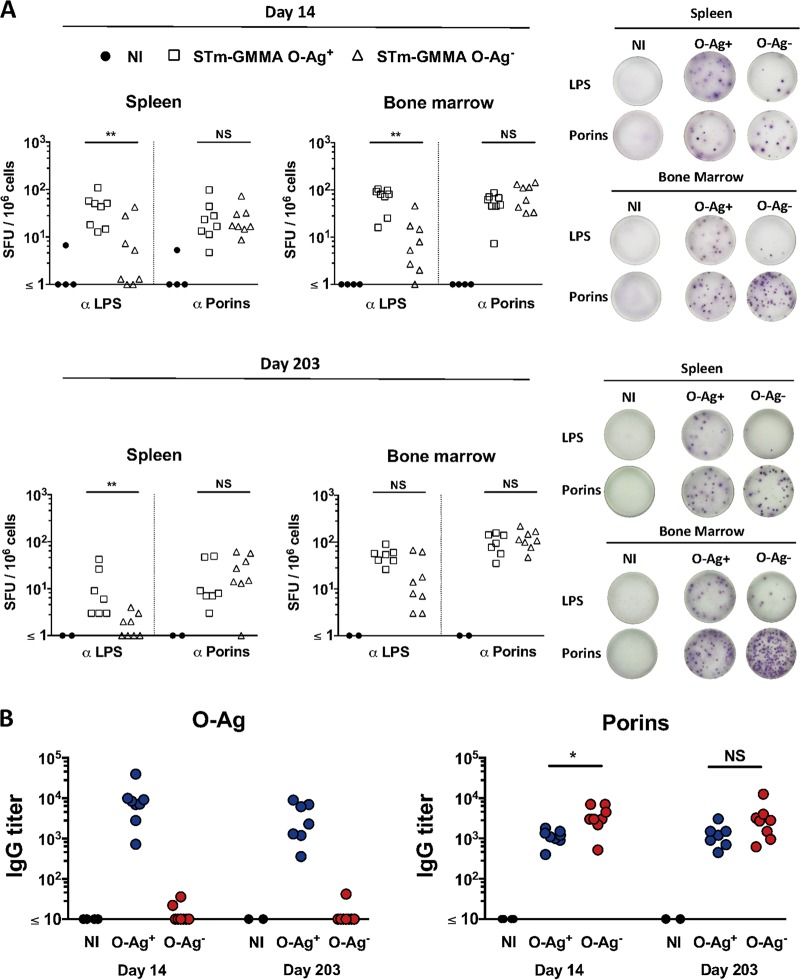
The persistence of Ab responses against LPS and porins following STmGMMA immunization. CD1 WT mice were immunized i.p. with 1 μg of STmGMMA O-Ag^+^ (open squares) or O-Ag^−^ (open triangles) at time zero and day 14. (A) B-cell ELISPOT assay used to assess IgG^+^ ASC against LPS and porins in spleen (left) and BM (right) 14 (top) or 203 (bottom) days after the second immunization. Splenocytes from each mouse were used to seed wells in triplicate at 5 × 10^5^/well. Numbers of spot-forming units (SFU) per million splenocytes are depicted in the graphs, and to the right are representative wells after development. Each dot represents one mouse. (B) ELISA used to analyze titers of IgG against O-Ag (left) or porins (right) in serum samples from mice immunized twice with either STmGMMA O-Ag^+^ (red circles) or O-Ag^−^ (blue circles) for the time periods indicated. Each circle represents one serum sample. The Mann-Whitney U test with Bonferroni correction for multiple comparisons was used to determine significant differences between groups linked by bars. NI, nonimmunized; NS, not significant;*, *P* ≤ 0.05; **, *P* ≤ 0.01.

A highly desirable feature of a vaccine is to induce responses that will persist. Therefore, we examined the response to porins and LPS in the spleen and bone marrow (BM) at later times after immunization. Furthermore, since O-Ag has been hypothesized to limit Ab binding to the Gram-negative bacterial surface, we assessed if removing O-Ag from GMMA could augment the Ab response to porins after immunization. Mice were immunized at time zero and boosted 14 days later with 1 μg of STmGMMA that were O-Ag replete (O-Ag^+^) or deficient (O-Ag^−^), and the response was examined 14 or 203 days later. Robust antigen-specific B-cell responses to LPS and porins were observed after immunization with O-Ag^+^ STmGMMA in both the spleen and the BM at days 14 and 203 (Fig. 5A). After immunization with O-Ag^−^ STmGMMA, there was not a dramatic change in the anti-porin B-cell response, and thus, the loss of O-Ag from GMMA is not sufficient to promote a lasting increase in the porin-specific response (Fig. 5A and B). There was a surprising response to LPS in mice that received O-Ag^−^ STmGMMA (Fig. 5A). Such antibodies are likely to target non-O-Ag and noncore regions of the LPS molecule since serology confirmed that there was a minimal response to the O-Ag and core oligosaccharide purified from *S*. Typhimurium in this group (Fig. 5B). Therefore, STmGMMA induce persisting Ab responses in the BM and spleen after immunization.

### The presence of O-Ag in STmGMMA enhances protection in the spleen.

**FIG 6:**
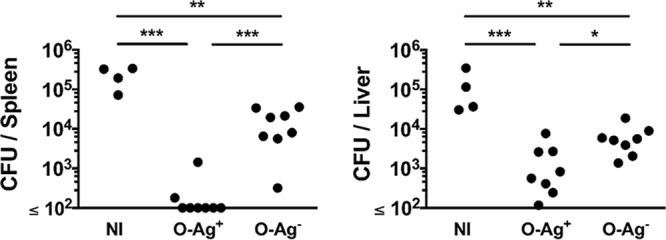
Immunization with O-Ag^−^ STmGMMA offers protection against infection in the spleen and liver. CD1 mice were immunized with STmGMMA O-Ag^+^ or O-Ag^−^ at time zero and day 14 and subsequently challenged i.p. with 2 × 10^4^ CFU of *S*. Typhimurium SL1344 at day 28. Bacteria in the spleen (left) and liver (right) were enumerated 3 days after infection. Each circle represents one mouse, and graphs are representative of two independent experiments. One-way ANOVA with Tukey’s multiple-comparison test were used to determine significant differences between groups linked by bars. NI, nonimmunized;*, *P* ≤ 0.05; **, *P* ≤ 0.01; ***, *P* ≤ 0.001.

It is clear from previous studies that Ab to O-Ag, generated after natural infection or after immunization with an O-Ag conjugate vaccine, can protect against infection (26, 30, 36, 37). Nevertheless, STmGMMA are known to contain other antigens, such as OmpD (33), that are targets of protective antibodies. Therefore, we tested the level of protection observed after immunization with O-Ag^+^ or O-Ag^−^ STmGMMA. Mice were immunized twice, 14 days apart, with the different STmGMMA and then challenged with *S*. Typhimurium, and the bacterial burdens were assessed 3 days later (Fig. 6). Mice immunized with both types of STmGMMA had bacterial burdens in the liver and spleen that were significantly lower than those of nonimmunized mice (Fig. 6). Nevertheless, mice immunized with O-Ag^+^ STmGMMA had markedly lower bacterial numbers in the spleen than mice immunized with O-Ag^−^ STmGMMA (typically, a >70-fold difference in mean bacterial numbers). Mice immunized with O-Ag^+^ STmGMMA also had lower bacterial burdens in the liver than mice that received O-Ag^−^ STmGMMA, but this difference was more marginal (typically, an ~10-fold difference in mean bacterial numbers). Thus, immunization with STmGMMA deficient in O-Ag can reduce infection in the spleen and liver, but the presence of O-Ag can enhance this protection and this effect is more pronounced in the spleen than in the liver.

## DISCUSSION

Previous work has shown that immunization with OMVs can protect against infection (1, 8, 10–17). Vaccines based on OMVs may offer an advantage over other strategies, such as O-Ag conjugates, through increased coverage of antigens in their natural conformation, their simplicity and cost of production, and their intrinsic adjuvant activity. In this study, we have shown that there is a strong dependence on B cells for the protection afforded by immunization with STmGMMA. Furthermore, while Ab induced by STmGMMA that lacks O-Ag is protective, the presence of O-Ag in the particles can bolster this protection, particularly in the spleen. Our evidence also suggests that the Ab response to STmGMMA is long-lived, as >200 days after immunization, there was a significant and persisting number of ASC specific for LPS and porins in the BM. B cells can provide Ab-dependent and -independent protection against infection. Nevertheless, in this instance, Ab is likely to make a major contribution, since opsonization of bacteria with serum samples from immunized mice is sufficient to reduce colonization and the absence of O-Ag reduces the protection afforded by immunization (37).

Loss of the immunodominant O-Ag (28, 30) from STmGMMA reduced the protection afforded by immunization, although bacterial burdens in the spleen and liver were still >20-fold lighter than those of nonimmunized controls. However, compared to O-Ag^−^ STmGMMA, the presence of O-Ag in STmGMMA was associated with a greater enhancement of protection in the spleen than in the liver. This suggests that the contribution of protective Ab to O-Ag varies for STmGMMA at different sites. Since Abs to *S*. Typhimurium largely protect through cell-dependent mechanisms in mice (38), it is likely that these differences reflect the types of immune cells present in various organs at different stages of infection (39). Other reasons for these differences include potentially the levels of O-Ag expression by bacteria entering these sites, the nature of the model, or other unappreciated reasons. Nevertheless, what is not clear is whether such differences are clinically meaningful, as it is not known what reduction in bacterial numbers is necessary to protect against disease. In reality, the level of protection needed to prevent disease is likely to depend upon the magnitude of the dose required to cause an infection within an individual, which will vary depending upon factors such as nutritional status, immune status, and geographic location. Since quantitative culture studies show that the bacteremia detected in patients with iNTS disease and HIV coinfection is low, in the order of a few bacteria per milliliter (40), it may mean that only modest levels of protection by a vaccine are needed. Indeed, the period between loss of maternal Ab and the first acquisition of IgG to NTS in infants is associated with an increased risk of invasive disease (24). Therefore, nonsterilizing immunity could be sufficient to provide widespread protection in vulnerable groups. The data in the present study indicate that Ab to surface antigens other than O-Ag can reduce bacterial numbers, and it is now necessary to better understand the nature of these protective antigens and if they can be manipulated to provide enhanced protection within a vaccine.

A single Gram-negative bacterium can contain millions of LPS molecules (41), and each O-Ag can contain over a hundred repeats (42), although in GMMA, estimates suggest that numbers of O-Ag repeats are lower than in WT bacteria (43). In contrast, estimates of the numbers of porin molecules expressed per cell suggest there is approximately 1 porin molecule for every 10 LPS molecules (44). Thus, epitopes for the O-Ag are likely to be present in a significant excess over those for porins. The combination of a higher epitope frequency and the spatial localization of O-Ag, being more distal from the cell surface than porins, makes the more rapid detection of switching to IgG against porins surprising. The level of response to an antigen can be important. In some individuals with HIV, atypically high titers of Ab to LPS O-Ag from *Salmonella* have been shown to inhibit bacterial killing in a serum bactericidal assay (31). Furthermore, a similar phenomenon has been observed in some patients with bronchiectasis and concurrent *Pseudomonas aeruginosa* infection, and in this case, the presence of inhibitory Ab correlates with worse disease (45). Therefore, examination of the Ab responses to STmGMMA with and without O-Ag offered an opportunity to examine whether O-Ag limited the development of Ab responses to other antigens. We expected there to be an increase in the response to porins in the absence of O-Ag since the access of B-cell receptors to these molecules on the bacterial surface could reasonably be expected to be enhanced. With the exception of a small augmentation of anti-porin Ab titers at day 14, we did not find this to be the case. This may be because responses to porins had already reached a peak level or because the *tolR* mutation can result in O-Ag being shorter in GMMA, meaning that anti-porin responses are readily induced anyhow (43), or because loss of *wbaP* influenced the frequency of LPS or porins in the STmGMMA (46). Moreover, the modest size of spherical STmGMMA particles may mean that O-Ag is not effective at occluding access to the surface of the GMMA. Nevertheless, it suggests that the absence of O-Ag from STmGMMA does not increase the frequency of the porin-specific Ab response.

IgM to LPS and porins was induced rapidly. When digested serum samples containing F(ab′)_2_, Fab, and Fv fragments of IgM were used in ELISAs, a reduced IgM signal, compared to that in undigested serum samples, was detected in anti-LPS ELISAs. This difference was greater in serum samples from mice immunized for 7 days than in samples from mice boosted with STmGMMA. Therefore, while IgM does not necessarily need to be in pentameric form to bind to these antigens, it is probable that the higher avidity of undigested IgM aids binding to LPS, particularly at earlier time points after immunization. Nevertheless, other factors may contribute to the degree of difference observed between digested and undigested serum samples. For instance, there may be fewer epitopes available on digested than nondigested IgM for the secondary Ab used in the ELISA to bind to, and this may alter the intensity of the signal detected. Further experiments are needed to understand fully the role of IgM avidity in Ab binding to these antigens. In contrast to IgM, the IgG response to porins and LPS developed with distinct kinetics after immunization with STmGMMA, despite both antigens being located in the same antigenic complex. IgG to porins was detectable at 4 days after infection, whereas IgG to LPS was not detectable until after a week. While some of this may reflect differences in germline affinities of antibodies (47), it was still surprising considering the rapid detection of IgM to both antigens and that LPS O-Ag is stoichiometrically the predominant antigen, with each individual O-Ag typically containing multiple repeats of the same epitope. Both LPS and porins have been reported to induce T-cell-independent responses when given as purified antigens (33, 48). Porin proteins, like OmpD, probably induce T-cell-independent responses through a combination of their capacity to oligomerize into units of trimers and their ability to interact with Toll-like receptor 2 (TLR2) and TLR4, and the latter property is lost if the proteins are digested with proteinase K (49). STmGMMA induced B1b-cell responses in WT and T-cell-deficient mice, and our previous work found that live *S*. Typhimurium and porins could induce responses in this B-cell subset (33). Antigens derived from pathogens that induce B1b responses are typically targets of protective immunity, and multiple antigens that induce B1b-cell responses, such as pneumococcal polysaccharide and the Vi capsular polysaccharide from *S*. Typhi, are used as vaccines in humans (35, 50). This reinforces the concept that identification of B1b antigens may be a helpful strategy to identify candidate antigens for vaccines.

The strong T-cell-independent response to porins within STmGMMA was associated with the early induction of IgG2b, IgG2c, and IgG3. Indeed, the only feature of the response dependent upon T cells was the induction of specific IgG1, an isotype we have reproducibly found to be poorly detected after infection with live *S*. Typhimurium (32). Moreover, in the response to live organisms, we and others have observed that T cells and molecules associated with classical T-dependent responses such as CD28, CD40-ligand, and ICOS (32, 33, 51, 52) are required for a significant IgG2a or IgG2b response. An additional difference between the responses to STmGMMA and live *S*. Typhimurium is the extensive early GC response, which is absent after live *S*. Typhimurium administration and something that live *S*. Typhimurium can actively suppress (47, 53, 54). Therefore, the cell surface antigens present in STmGMMA cannot be sufficient on their own to be responsible for these differences and the suppression of early GC formation observed after live infection. It is possible that the T-cell-independent switching to IgG2a seen after immunization with STmGMMA reflects more what is seen after infection with viruses and virus-like particles (55).

The speed of induction of the GC seen after STmGMMA was striking and resembles that observed after immunization of mice with the T-cell-independent antigen NP-Ficoll, where there is a high frequency of antigen-specific B cells (56). A recent study showed that T-cell-independent switching to IgG against a peptide antigen within liposomes was strictly dependent on concomitant signaling via TLR4 and TRIF (57). Therefore, the multiple TLR4 ligands found in STmGMMA could modulate the response to other antigens within the complex. This means that ultimately the Ab response to the same antigen can vary dramatically, depending upon the context in which the antigen is encountered, for instance, whether the antigen is part of a viable organism, in a complex with other antigens, or entirely in isolation. Indeed, we have previously shown that the pattern of IgG switching to flagellin from *S*. Typhimurium varies dramatically, depending upon whether mice encounter flagellin as a purified molecule or in its native context, attached to the surface of a live organism (51). An appreciation of how to control the direction of IgG switching to an antigen will help us understand how to maximize the potential of OMVs and GMMA as platforms for vaccine delivery.

## MATERIALS AND METHODS

### STmGMMA production, purification, characterization, and electron microscopy.

Hyperblebbing GMMA-producing strains (provided by the GSK Vaccines Institute for Global Health) and the production and purification of GMMA were performed as described previously (3, 6, 58). In brief, the *tolR* gene in *S*. Typhimurium isolate 1418 (59) was replaced with the kanamycin resistance gene *aph* (3, 6, 43). For the generation of a GMMA-producing strain lacking the O-Ag, *wbaP* was replaced with the chloramphenicol resistance cassette (*cat*). Forward and reverse primers with approximately 50 bp homologous to upstream and downstream regions of the gene to be deleted and approximately 20 bp corresponding to flanking regions of the respective resistance cassette were used to amplify replacement constructs. The primers used for *aph* resistance replacement are reported elsewhere (6), and the primers used for *cat* resistance replacement were 5′ CGCAGGCTAATTTATACAATTATTATTCAGTACTTCTCGGTAAGCGTGTAGGCTGGAGCTGCTTCG 3′ and 5′ CTTAATATGCCTATTTTATTTACATTATGCACGGTCAGAGGGTGACATATGAATATCCTCCTTAG 3′. Amplicons were then purified and transformed into *S*. Typhimurium 1418 with the lambda phage recombinant system (*red* operon) located on pAJD434 as described in reference 3. A specific anti-O:4 LPS O-Ag MAb (provided by the GSK Vaccines Institute for Global Health) was used to confirm the loss of O-Ag from STmGMMA generated from this strain (25). To purify GMMA, 300 ml of fresh LB medium was inoculated with bacteria from an overnight culture to generate a culture with an optical density at 600 nm (OD_600_) of 0.05. Once the culture reached an OD_600_ of 0.7 to 1, it was incubated for a further 3 h and then centrifuged for 10 min at 3,220 × *g*. The supernatant containing GMMA was filtered through a 0.22-μm filter and ultracentrifuged for 2 h at 186,000 × *g* with a 45 or 70 Ti rotor (Beckman Coulter, Inc.). GMMA were then resuspended in phosphate-buffered saline (PBS), filtered through a 0.22-μm filter, and stored at 4°C until use. The total protein concentration of GMMA samples was assessed by Lowry assay (Bio-Rad). For TEM, STmGMMA were fixed in 2.5% glutaraldehyde–0.1 M phosphate buffer and embedded in Epon-araldite resin, and 70- to 80-nm sections were cut and stained with uranyl acetate and Reynolds lead citrate before being visualized with a JEOL 1200EX at the Centre for Electron Microscopy at the University of Birmingham.

### Mice, immunizations, and bacterial challenge strains.

All of our animal studies had ethical approval from the University of Birmingham and UK Home Office approval and were performed under project license 30/2850 in compliance with the Animals (Scientific Procedures) Act 1986. Inbred WT C57BL/6 males 6 to 8 weeks old or outbred WT CD1 females 6 to 8 weeks old were obtained from OLAC Harlan. In experiments, C57BL/6 mice were used as WT mice unless otherwise stated. TCRβδ^−/−^ and IgH^−/−^ (33, 60) mice on a C57BL/6 background were bred at the Biomedical Service Unit, University of Birmingham. Mice were immunized via i.p. injection with STmGMMA O-Ag^+^ or O-Ag^−^ (the same 1-μg dose of protein was given in all of the experiments in this study) diluted in 200 μl of PBS. Analysis showed that the ratio of O-Ag to protein in STmGMMA O-Ag^+^ preparations was in the range of 0.7 to 0.9 part O-Ag to 1 part total protein (wt/wt). For protection studies, *S*. Typhimurium attenuated AroA^−^ strain SL3261 (described in reference 32) and virulent strain SL1344 were used at infection doses of 5 × 10^5^ and 2 × 10^4^ CFU, respectively, and administered i.p. in PBS. Opsonization experiments were performed as described in reference 32, with multiple nonpooled serum samples from nonimmunized mice or mice that had been immunized with STmGMMA for 35 days. Prior to use, serum samples were heated for 30 min at 56°C to inactivate complement. Infection doses of *S*. Typhimurium SL3261 and serum samples (1:100) were then mixed for 30 min at room temperature before infection. Bacterial viability and lack of agglutination in the presence of opsonizing Ab were confirmed by plating bacteria onto agar and ensuring that they grew as discrete colonies at the appropriate frequency. Bacterial numbers were determined by direct culturing of homogenized spleen and liver samples.

### ELISA and ELISPOT assay.

Antigens used in ELISAs and ELISPOT assays were STmGMMA O-Ag^+^, porins (OmpC, OmpF, and OmpD) purified from *S*. Typhimurium (described in references 33 and 61), TLR grade *S*. Typhimurium LPS (Axxora ALX-581-011-L002), or O-Ag (with core sugars attached) purified from *S*. Typhimurium 1418 (provided by the GSK Vaccines Institute for Global Health and described in reference 62). All antigens were used at 5 μg/ml, and ELISAs were performed as previously described (32). Mouse serum was added to wells and then serially diluted, and the presence of bound Ab was detected with isotype-specific alkaline phosphatase (AP)-conjugated goat anti-mouse Abs (Southern Biotech) and developed with Sigma FAST *p*-nitrophenylphosphate tablets. Titers were calculated by identifying the dilution at which the signal reached a set OD_405_.

To obtain splenic cells for ELISPOT assay and flow cytometry, spleens were disrupted through a 70-μm cell strainer into Roswell Park Memorial Institute (RPMI) 1640 medium (Gibco) supplemented with 10% heat-inactivated fetal bovine serum and 5% penicillin-streptomycin (complete RPMI). BM cells were obtained by flushing one hind leg (femur and tibia) per mouse with RPMI 1640 medium. Erythrocytes were lysed with ammonium-chloride-potassium lysis buffer (Gibco). Single-cell suspensions were washed in complete RPMI 1640 medium and resuspended to the desired concentrations. For ELISPOT assay, multiscreen filter plates (Merck Millipore) were coated with antigen diluted in PBS overnight at 4°C, washed with PBS, and then blocked with complete RPMI 1640 medium for 1 h at 37°C. The wells were then washed once with PBS, and 5 × 10^5^ cells from spleen or BM were seeded per well in 200 μl of complete RPMI 1640 medium, each sample in triplicate. Plates were then incubated for 6 h at 37°C in 5% CO_2_. After incubation, cells were lysed and washed away with PBS–0.05% Tween. Bound mouse Ab was detected with goat anti-mouse Ab linked to AP (Southern Biotech) diluted in PBS overnight at 4°C. The following day, wells were washed three times with PBS–0.05% Tween and once with PBS. Spots were developed with Sigma FAST 5-bromo-4-chloro-3-indolylphosphate (BCIP)–Nitro Blue Tetrazolium tablets (Sigma) diluted in sterile water in accordance with the manufacturer’s instructions.

### Depletion of IgM from serum samples and digestion of serum samples to generate IgM F(ab′)_2_, Fab, and Fv.

Monoclonal rat anti-mouse IgM (clone 1B4B1; Southern Biotech) was conjugated to Sepharose 4B (GE Healthcare) in accordance with the manufacturer’s protocol. Two hundred microliters of bead slurry was mixed with 200 μl of serum for 15 min. Beads were centrifuged at low speed. The supernatant, containing depleted serum, was compared with undepleted serum by ELISA to assess IgG and IgM contents and antibodies specific for LPS and porins. Relative levels of IgM and IgG in the serum samples were assessed by ELISA by the basic technique described above but with the following differences. The capture Ab in the ELISAs was the rat anti-mouse IgM used as described above or unlabeled sheep anti-mouse IgG (both used to coat plates at 1 μg/ml), and secondary antibodies were HRP-conjugated goat anti-mouse kappa light chain (Millipore) for IgM and goat anti-mouse heavy and light chains (Southern Biotech) for IgG. Color was developed with 3,3′5,5′-tetramethylbenzidine dihydrochloride tablets (Sigma) and H_2_SO_4_ in accordance with the manufacturer’s instructions. The OD_450_ was measured.

IgM F(ab′)_2_, Fab, and Fv fragments from serum samples were generated by adapting a previously described method (63). Serum samples were diluted with an equal amount of 20 mM sodium acetate, pH 4.5, and adjusted to a pH of 4.0 with HCl. Immobilized pepsin (Thermo Scientific) was then added to these serum samples at a ratio of resuspended enzyme beads to serum of 1:50, and the mixture was incubated at 37°C with shaking for 24 h. The reaction was stopped by removal of the pepsin beads after centrifugation at 1,000 × *g* for 5 min. Digestion of IgM into F(ab′)_2_, Fab, and Fv fragments was verified by SDS-PAGE. Serum samples were then diluted in PBS, and the pH was confirmed to be in the range of 7 to 7.5 before they were used in ELISAs.

### Fluorescence-activated cell sorter (FACS) analysis.

Cells from spleen or peritoneal flushes were stained with one or more of the following anti-mouse MAbs: CD3-fluorescein isothiocyanate (FITC), CD3-phycoerythrin (PE)/Cy7 (145-2C11), GL7-eFluor 450 (GL7), F4/80-FITC (BM8), CD4-eFluor 450 (RM4-5), CD44-peridinin chlorophyll protein (PerCP)-Cy5.5 (IM7), PD-1–PE (J43), IgM-PE-Cy7 (11/41), and CD5-PerCP-Cy5.5 (53-7.3) from EBioscience; B22 0PE (RA3-6B2), CD95-PE-Cy7 (Jo2), CD19-allophycocyanin (APC)-Cy7 or -APC (ID3), CD21-FITC (7G6), and CD23-PE (B3B4) from BD Pharmingen; B220BV510 (RA3-6B2) and CD138-PE (281-2) from BioLegend; and IgG2b-FITC and IgG2c-FITC (polyclonal) from Southern Biotech. Rat anti-mouse CXCR5 (551961) (BD Pharmingen) was detected with an Alexa Fluor 647-conjugated anti-rat antibody (712-606-153) from Jackson ImmunoResearch, Inc. Cells were then assessed on a CyAn ADP flow cytometer and analyzed with FlowJo 9.3.2. (TreeStar). For intracellular staining, cells were first stained for surface markers and then fixed and permeabilized with Cytofix/Cytoperm Plus (BD Biosciences).

### Immunohistology.

Spleens were processed and stained as described previously (32). In short, spleens were frozen in liquid nitrogen and 5.5-μm-thick sections were cut and allowed to dry before fixation in acetone for 20 min at 4°C. In all cases, antibodies were added for 45 min of incubation at room temperature. Sheep anti-mouse IgD (Abcam) was detected with peroxidase-labeled donkey anti-sheep antibody (Jackson ImmunoResearch, Inc.) and developed with 3,3′-diaminobenzidine tablets (Sigma). CD3 and peanut agglutinin (PNA) were detected with rat anti-mouse CD3 (AbD Serotec), biotinylated rabbit anti-rat (Dako), and biotinylated rabbit anti-PNA (Vector Laboratories) antibodies. Biotinylated Ab binding signal was developed by using a streptavidin-biotinylated AP complex (Vectastain; Vector Laboratories) with Naphthol AS-MX phosphate, levamisole, and Fast Blue Salt.

### Statistical analyses.

Statistical tests were performed with Prism software. The tests performed are indicated in the legends but included the Kruskal-Wallis test with Dunn’s multiple-comparison test, the two-tailed Mann-Whitney U test (with Bonferroni correction for multiple comparisons if required), and one-way analysis of variance (ANOVA), followed by Tukey’s multiple-comparison test. *P* ≤ 0.05 was considered significant.
