# Supplementary figures and images for: IgG Responses to Porins and Lipopolysaccharide within an Outer Membrane-Based Vaccine against Nontyphoidal Salmonella Develop at Discordant Rates
Source: mBio. 2018 Mar 6;9(2):e02379-17. doi: 10.1128/mBio.02379-17 (PMC5844998; doi:10.1128/mBio.02379-17)

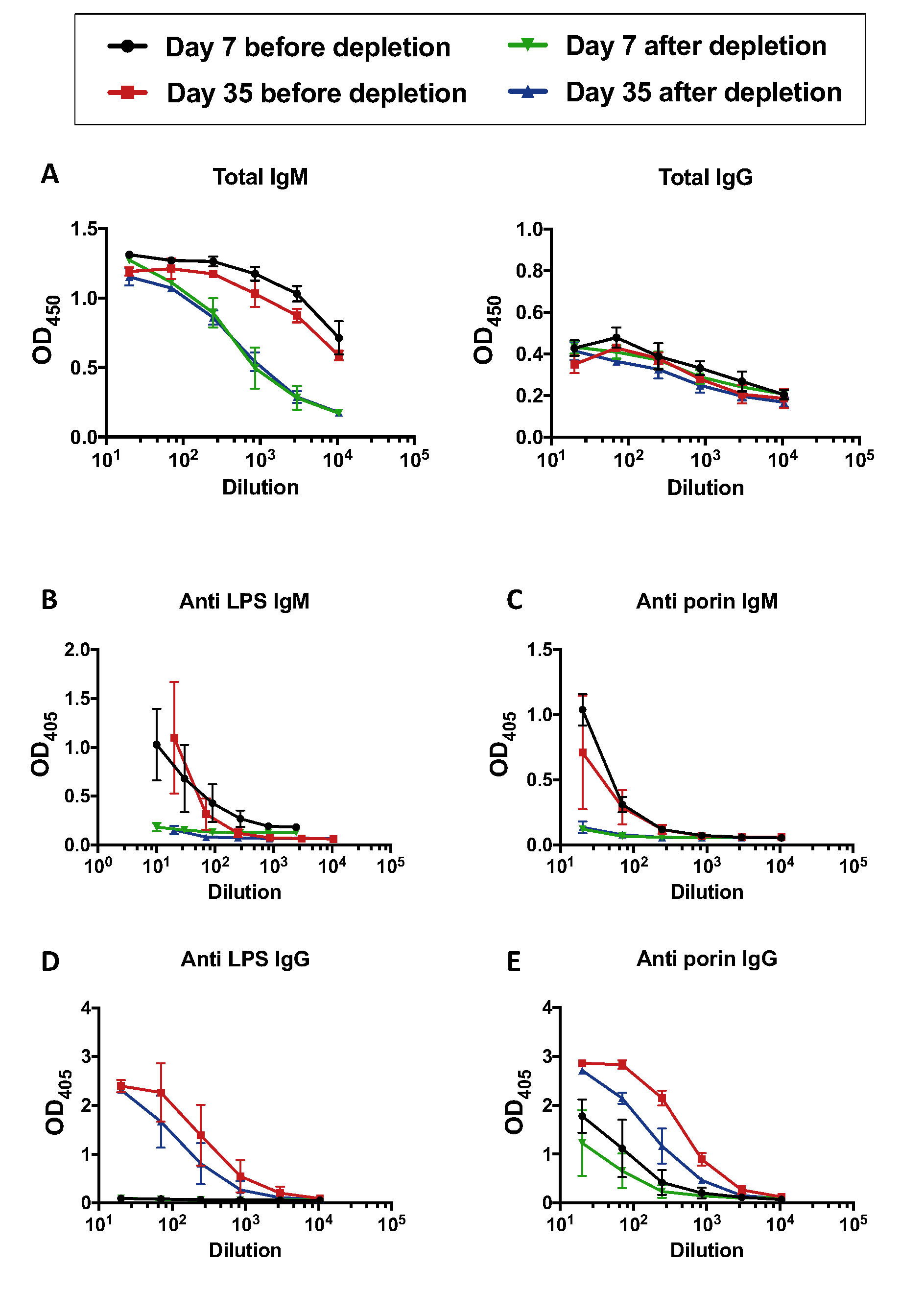

Supplement: FIG S1 [file mbo001183757sf1.tif]

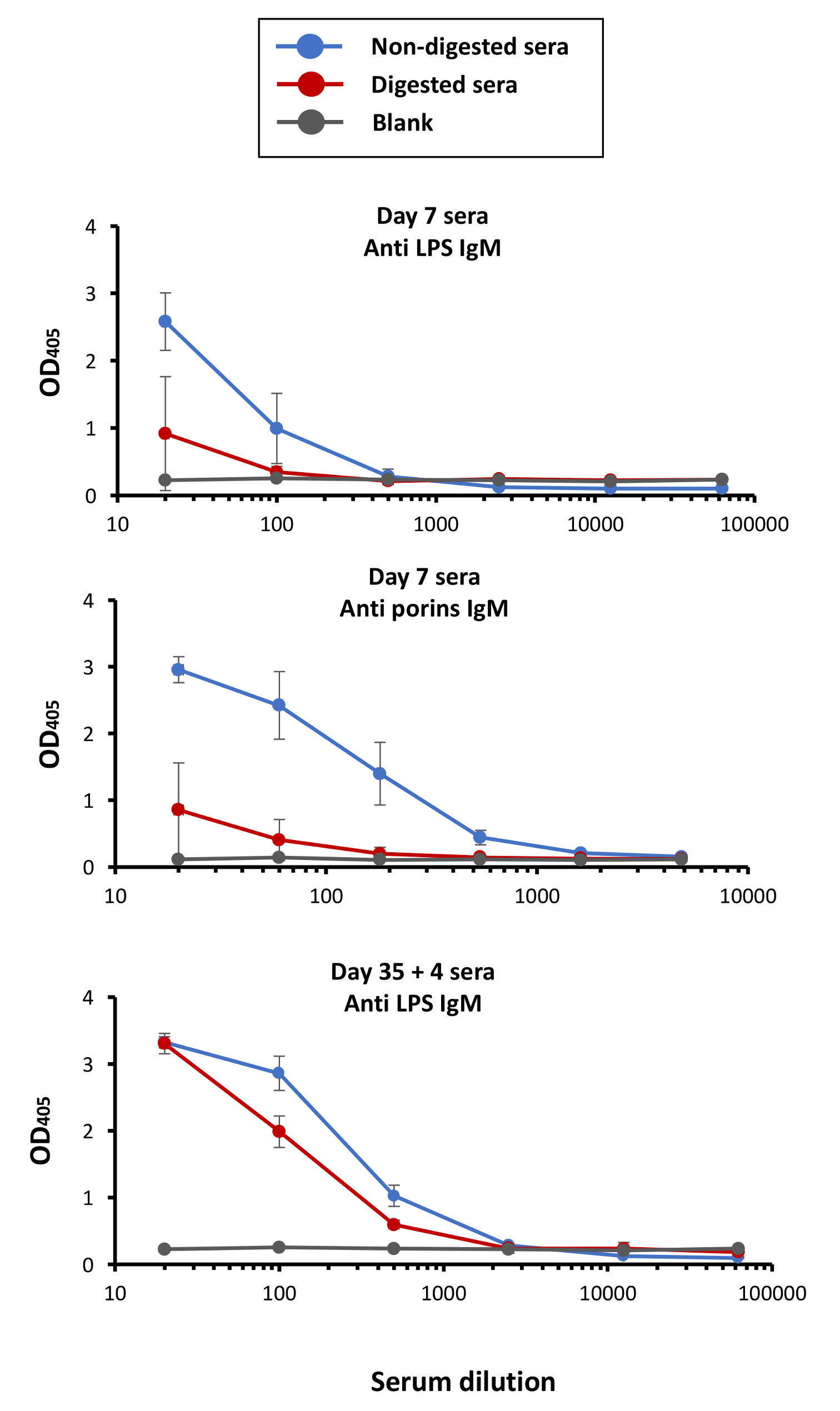

Supplement: FIG S2 [file mbo001183757sf2.tif]
